# Small Cell Carcinoma of the Larynx Treated by Concurrent Chemoradiotherapy: A Case Report

**DOI:** 10.1155/2012/316165

**Published:** 2012-03-29

**Authors:** Susumu Nakahara, Norihiko Takemoto, Hidenori Inohara

**Affiliations:** ^1^Department of Otorhinolaryngology-Head and Neck Surgery, Osaka University Graduate School of Medicine, 2-2 Yamadaoka, Suita, Osaka 565-0871, Japan; ^2^Department of Otolaryngology, Suita Municipal Hospital, 2-13-20 Katayama, Suita, Osaka 564-0082, Japan

## Abstract

Small cell carcinoma (SmCC) generally occurs in the lung and extrapulmonary SmCC is a relatively rare entity. Here, we present a case of a 42-year-old male with SmCC of the larynx diagnosed as cT3N0M0. Concurrent chemoradiotherapy using cisplatin (CDDP) and etoposide (VP-16) was given, which achieved complete response (CR). Lung metastasis became evident in 16 months whereas the locoregional control remained good. In spite of intensive chemotherapy using CDDP and irinotecan (CPT-11), the patient died of disease in 34 months after the first interview. Since the prognosis of laryngeal SmCC is extremely poor, organ preservation therapy should be considered instead of radical laryngectomy.

## 1. Introduction

Small cell carcinoma (SmCC), one of neuroendocrine malignant neoplasms, occurs predominantly in the lung. SmCC shows aggressive clinical progression, resulting in poor prognosis [1]. Primary SmCC in the extrapulmonary region was first reported in 1930 [2], and since then, SmCC has been shown to occur in a diversity of locations throughout the body. Extrapulmonary SmCCs constitute 2.5–5% of all SmCCs [3].

In the head and neck region, the larynx is the most common site affected by SmCC, but SmCC accounts for only 0.5% of all laryngeal malignancies [4]. SmCC preferably develops in the supraglottic area of the larynx [5]. Laryngeal SmCC pursues a mortal course as the same as pulmonary SmCC. It is reported that 2-year and 5-year survival rates of laryngeal SmCC are no more than 16% and 5%, respectively [5]. Most patients with laryngeal SmCC are heavy smokers with a median age of about 64 years, and patients younger than 40 years are rare [6].

 We present an additional case of primary SmCC of the larynx which affected a relatively young male. Although concurrent chemoradiotherapy was given and the larynx was successfully preserved, the patient died of pulmonary metastasis.

## 2. Case Summary

**Figure 1:**
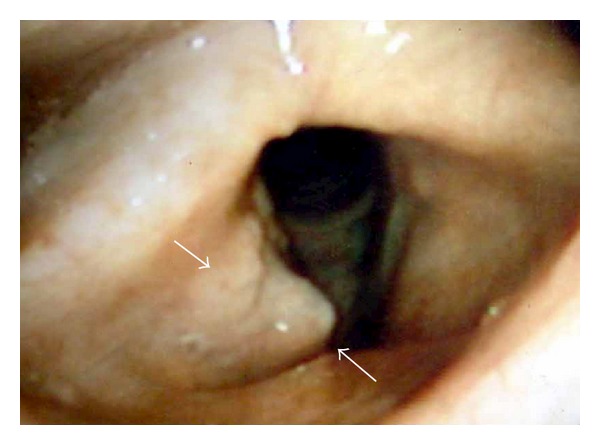
Flexible laryngoscopy showing an obtrusive tumor localized in the right false cord (arrows).

**Figure 2:**
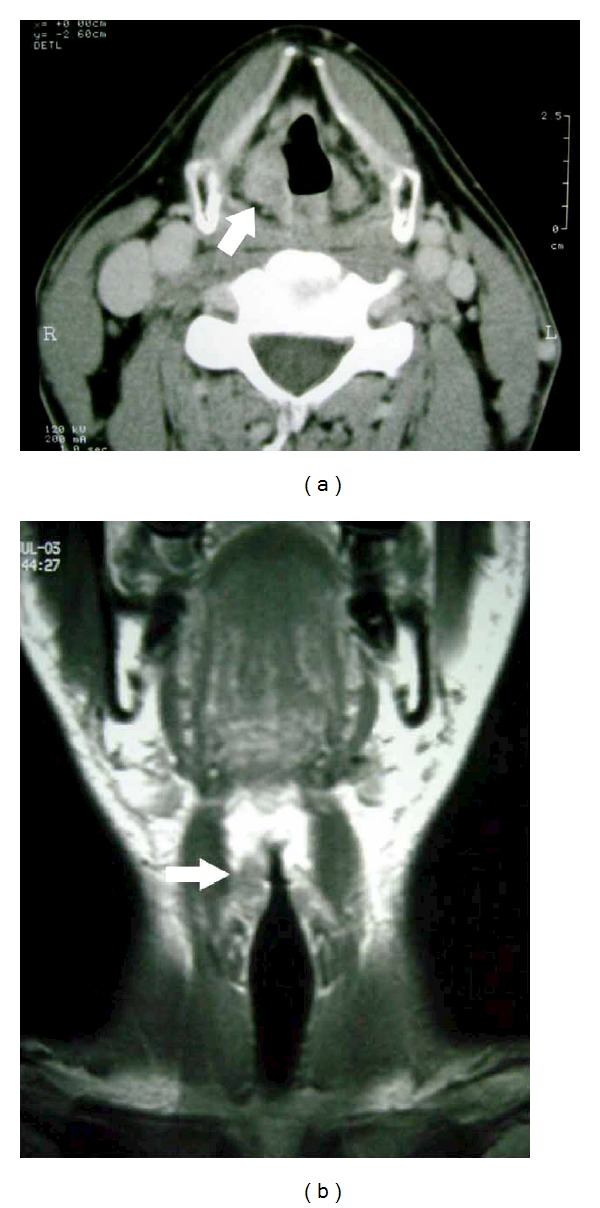
CT-scan (a) and MRI (b) showing a swelling of the right false cord (arrows) with involvement of the paraglottic space and without a sign of significant lymphadenopathy.

**Figure 3:**
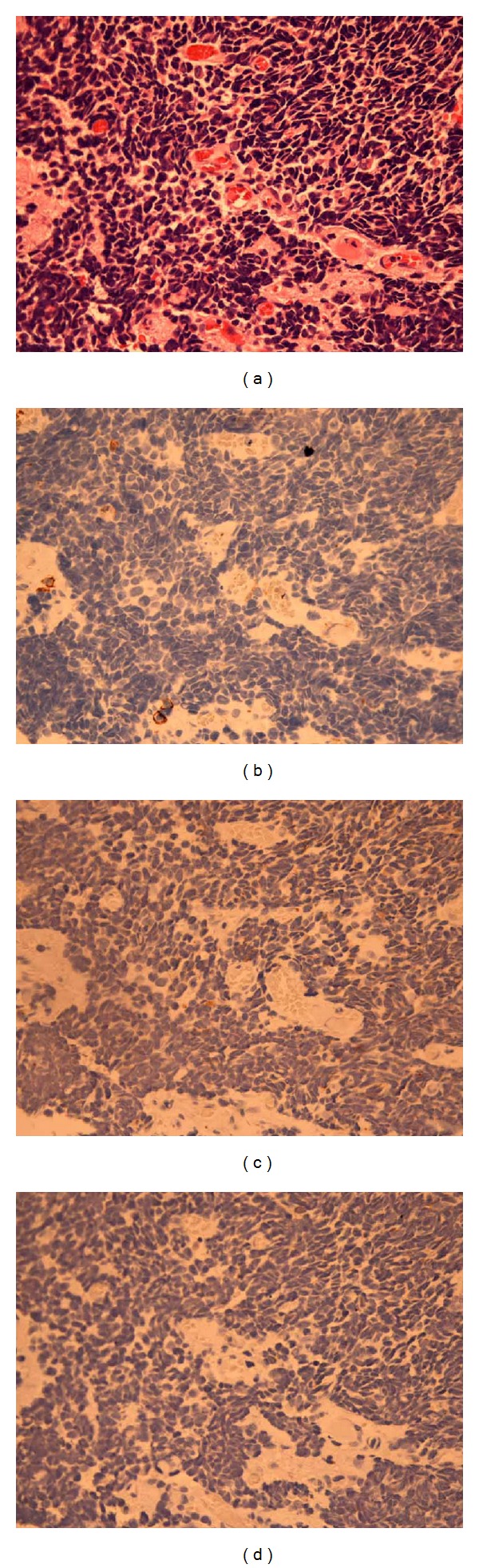
Histopathological examination by HE staining (a) reveals cluster of undifferentiated small cells. Immunohistochemical analyses of LCA (b), EMA (c), and chromogranin A (d) show no significant positive staining (original magnification; ×400).

A 42-year-old male, who had complained of sore throat for 5 months, was presented to our hospital in July 2003 with a 5-month history of sore throat. He had a long history of cigarette smoking, and his Brinkman's index reached 440. Flexible laryngoscopy revealed a conspicuous lesion localized in the right false cord (Figure 1), and the vocal fold movement was limited. Computerized tomography (CT) and magnetic resonance imaging (MRI) showed swelling of the right false cord without significant cervical lymphadenopathy and demonstrated that the paraglottic space was involved with no sign of thyroid cartilage erosion (Figure 2). F-18-fluorodeoxyglucose positron emission tomography (FDG-PET) identified no other active lesion than the larynx. Histological examination of a biopsied specimen revealed sheets of undifferentiated small atypical cells with minimal cytoplasm (Figure 3(a)), which is similar to the characteristic of pulmonary SmCC. Immunohistochemical analyses revealed that neoplastic cells did not express leukocyte common antigen (LCA), epithelial membrane antigen (EMA), and chromogranin A (Figures 3(b), 3(c), and 3(d)). Taken these findings into consideration, the final diagnosis was made to be primary SmCC of the larynx classified as cT3N0M0.

The patient received 2 cycles of systemic chemotherapy with cisplatin (CDDP) (80 mg/m^2^, day 1) and etoposide (VP-16) (100 mg/m^2^, days 1–3) at 4-week interval and concurrent radiotherapy. Radiotherapy was given by conventional fractionation at 2 Gy/fraction/day with a total dose of 66 Gy. There was no apparent acute toxicity except mucositis grade 2 around the larynx. After the concurrent chemoradiotherapy, he successfully attained complete remission and had remained free of recurrence for 16 months. In February 2005, he complained of prolonged cough and underwent CT scanning, which unveiled multiple pulmonary lesions. Transbronchial biopsy showed SmCC, suggesting lung metastasis from the primary laryngeal SmCC. He then received 3 cycles of another systemic chemotherapy with CDDP (60 mg/m^2^, day 1) and irinotecan (CPT-11) (60 mg/m^2^, day 1, 8, 15) at 3-week intervals according to the regimen for pulmonary SmCC. Although the metastatic lesions showed partial response for once, the residual gradually increased and he died of disease in May 2006.

## 3. Discussion

Since SmCC arising in the head and neck was first described in 1972 [7], SmCC has been reported to occur in multiple sites throughout the head and neck including the paranasal sinuses, oral cavity, salivary glands, pharynx, larynx, and thyroid gland [6]. According to a previous review, there were only about 160 cases of laryngeal SmCC reported in the literature prior to 1998 [8], whereas accumulating cases of laryngeal SmCC have been reported in the last decade. Laryngeal SmCC is not an extremely rare entity as once believed but is rather relatively infrequent so that it accounts for approximately 0.5% of all laryngeal malignancies [4].

The pretherapeutic definitive diagnosis is absolutely important to deliver an appropriate therapy to laryngeal SmCC. In particular, other neuroendocrine neoplasms of the larynx such as carcinoid, large cell neuroendocrine carcinoma, and paraganglioma should be clearly excluded [9]. Surgical resection such as total laryngectomy, which causes a severe decrease in quality of life, is not recommended as a first treatment of choice, because laryngeal SmCC is a lethal entity with an extremely poor prognosis [6]. Histological and immunohistochemical analyses by experienced pathologists are essential to distinguish SmCC from epithelial tumors or other neuroendocrine neoplasms [9]. In our case, LCA and EMA were immunohistochemically negative, excluding the possibility of lymphoma and differentiated epithelial carcinoma, respectively. In addition, the tumor was immunohistochemically negative for neuron-specific chromogranin A. This seems unusual for neuronendocirne-derived SmCC however, there have been several reports showing negative immunostaining of chromogranin A in extrapulmonary SmCCs [10]. Otolaryngologists as well as pathologists should keep it in mind that some SmCCs do not immunohistochemically express chromogranin A.

Recommendation for the primary treatment of laryngeal SmCC is based on that of pulmonary SmCC. At present, radiotherapy in combination with adjuvant or concurrent chemotherapy seems the primary treatment of choice by general consent. Systemic chemotherapy is advisable against laryngeal SmCC even at early stage, because it harbors the risk of occult metastases [6]. Recently, platinum-based chemotherapy regimens have become the mainstay of treatment [6]. In extensive or recurrent extrapulmonary SmCCs, platinum-based regimen showed a response rate of 72% and median survival duration of 8.5 months, whereas doxorubicin-based regimen resulted in 57% and 4.5 months, respectively [11]. In particular, the combined use of cisplatin and etoposide for neuroendocrine carcinomas of the head and neck has been reported to yield a high response rate [12]. Although concurrent chemoradiotherapy clearly improves response rate of primary lesion, the survival rate remains poor due to distant metastases [6]. The extent of disease at diagnosis represents the most sensitive predictor of survival [11]. We employed concurrent chemoradiotherapy using cisplatin and etoposide to treat the patient with laryngeal SmCC. Complete response was achieved with the larynx successfully preserved; nevertheless lung metastasis shortened his life.

Initial systemic work-up is essential in order to evaluate whether extrapulmonary SmCC of interest is primary or metastatic from the lung. There are a number of reports showing that SmCCs, which had been considered to originate primarily in the head and neck, turned out to be a metastasis from the lung [13]. In our case, once histological diagnosis of the laryngeal tumor was made as SmCC, we thoroughly examined by use of FDG-PET and whole body CT scanning to fix the lesion as primary or metastatic SmCC. There was no sign underlying a pulmonary active lesion before treatment. Unfortunately, we did not examine the expression of TTF-1 by immunohistochemical analysis, which is used to distinguish the tumor primary between lung and nonlung origin; however, we believe that the laryngeal SmCC is primary and the recurrent pulmonary SmCC is metastatic.

In conclusion, laryngeal SmCC is a relatively rare entity with high malignant potential. Clinicopathological work-up is essential to make a pretherapeutic definitive diagnosis of SmCC and to distinguish primary from metastatic SmCC. Combined chemoradiotherapy aiming at organ preservation is preferred because most patients cannot survive despite all current attempts at treatment.
